# Predictable or Not? Individuals’ Risk Decisions Do Not Necessarily Predict Their Next Ones

**DOI:** 10.1371/journal.pone.0056811

**Published:** 2013-02-21

**Authors:** Kin Fai Ellick Wong, Cecilia Cheng

**Affiliations:** 1 Department of Management, Hong Kong University of Science and Technology, Hong Kong Special Administrative Region, China; 2 Department of Psychology, The University of Hong Kong, Pokfulam, Hong Kong Special Administrative Region, China; University College London, United Kingdom

## Abstract

This research examines the extent to which people may be free to make choices by testing their consistency in choosing risk options. In two experiments, participants were instructed to make the “same” type of risk decisions repeatedly. Experiment 1 showed that when the information for decision is positively framed in terms of gain, the participant’s choice in a particular decision could not be predicted by his or her choice in another decision (*R^2^*s<.02). Experiment 2 showed a statistically significant predictability when the information is negatively framed in terms of loss, although the predictability was still very low (*R^2^*s<.07). These findings indicate the existence of a large room of variations in which a person may freely choose.

## Introduction

Scientific studies of human choice behaviors are paradoxical. On the one hand, scientists identify systematic patterns and regularities of choice behavior [1], [2]. On the other hand, if choice behavior is determined by these patterns and regularities, a choice will no longer be a choice because people “respond to” but do not “choose” it. This paradox stems from a more fundamental debate between determinism versus free will: whether human behavior is determined and automatic [3]–[5], or whether people are the sole agents who exercise control on at least some of their behaviors [6]–[9]. As an attempt to address this controversy, the present report describes two experiments that provide some empirical evidence that choice behavior may not be completely predictable, suggesting the possible existence of free will.

### Foundation of Testing Free Will

Scientists from multiple disciplines have shown interest in designing methods for testing whether or not people possess their own freedom to make choices [3], [10]–[12]. Despite considerable debates and controversies, a foundation emerges that involves the following set of propositions. First, free will is generally defined as something that “we could often have done otherwise than we in fact did” [13] or “could have done otherwise” [14]. This definition implies that at least certain human behaviors are not pre-determined, and that behavior is “more than the unavoidable consequences of genetic and environmental history of individual and possible stochastic laws of nature” ([4], p. 4500). Specifically, after a person has made a choice, if he or she is allowed to return to the moment right before making the decision, and if everything else is exactly identical to the previous decisional context, free will is evident when the person is able to make a different choice.

Second, based on the above understanding, to allow freedom, some physical and behavioral components cannot be summarized nor predicted by rules and laws. In the physical world, quantum physics offers a possible way for delineating the unpredictability of physical nature [15]. Similarly, the prerequisite of free will is that certain behaviors should contain some degree of unpredictable variability [6], [8].

It is important to note that our goal of this research is not to confirm the existence of free will. Our goal is to provide some evidence for the necessary, but not sufficient, room for the existence of free will. Demonstrating the unpredictability of behavioral variability does not necessarily reveal the existence of free will because such unpredictability may be due to a number of factors, such as randomness,variability in information sampling but not responding (e.g., as what the signal detection theory tells us), and/or other hidden factors [4]. Nonetheless, showing the unpredictability of behaviors is the necessary (though insufficient) condition for the existence of free will [4]. Unpredictability is the necessary evidence because it is the only part that distinguishes determinism from non-determinisms. Unpredictability is not the sufficient evidence because there could be unobserved factors contributing to the unpredictability.

### Free Will in Invertebrates

Brembs [8] reviewed a body of literature on invertebrates that supports the notion that invertebrate behavior is sufficiently unpredictable, fulfilling the prerequisite of freedom to choose their behavior. Studies on the variations of *Drosophila* behaviors [16]–[18] are of particular relevance to the present research. In the description of his well-known phototaxis experiments, Benzer observed that

“…if you put flies at one end of a tube and a light at the other end, the flies will run to the light. But I noticed that not every fly will run every time. If you separate the ones that ran or did not run and test them again, you find, again, the same percentage will run. But an individual fly will make its own decision.” (cited from [8], p. 934)

These findings indicate that although the light determines how likely each fly will move toward the light, each fly might still have some freedom to choose to fly or to stay. Otherwise, if each fly’s choice to move is determined by environmental and genetic factors, the flies that choose to move in a previous trial should choose to move again, and the flies that choose to stay should choose to stay again.

A later study by Quinn et al. [17] obtained a similar pattern of results. A group of flies learned to avoid one of two odors. In the first trial, the flies were tested with an odor. A certain percentage of the flies avoided the odor, but the remaining did not. The flies were then separated into two groups: the avoider group and the non-avoider group. In the second trial, these two groups were tested with the same odor again. A certain percentage of flies from the avoider group and a certain percentage of flies from the non-avoider group avoided the odor. Interestingly, the percentage of the avoiders in the two groups was roughly similar, which also approximated the proportion of the two groups in the first trial. Although learning about the odor determines how likely each fly will avoid the odor, each fly may still have some freedom to choose to avoid the odor or not. If the flies could not have done otherwise, each fly’s first choice should reliably predict its second choice.

### The Present Study

Following the rationale of the aforementioned studies on *Drosophila*, we examine the extent to which human choice behavior involves variability as the prerequisite of free will. In the two experiments, we instructed participants to make the “same” type of decisions repeatedly. The decisions are adapted from Tversky and Kahneman’s vignettes [2] in which the participant is asked to choose either a decision with a certainly less attractive outcome or an uncertainly more attractive outcome. When making this kind of decisions, robust findings revealed that people are generally risk averse when the vignettes are described in terms of gain (i.e., favoring “a certain gain of $50” to “a 50% chance of gaining $ 100 and a 50% chance of gaining nothing”), and that they are generally risk seeking when the vignettes are described in terms of loss (i.e., favoring “a 50% chance of losing $100 and a 50% chance of losing nothing” to “a certain loss of $50”).

If human choice behavior is largely determined, a person who chooses a “risky” option in one trial is likely to choose the “risky” option again in the other trial. On the other hand, if the person could have done otherwise, the person’s choice made in a particular trial may not be predicted by his or her choice in another trial.

#### Ethics

We declare that individual participants in the current study (Experiments 1 and 2) gave their written informed consent. The Institutional Review Boards at the University of Hong Kong approved the study.

## Experiment 1

### Methods and Materials

In Experiment 1, participants (*N* = 105) were asked to make a series of choices as if they were making it for real. They made two monetary decisions that were separated by a medical decision (see below), with the order of the two monetary decisions counterbalanced across participants. As the participants were residing in Hong Kong, we changed the currency to Hong Kong dollars in the monetary decisions so that participants were more familiar with the context of these decisions. The percentage of participants who made each choice is presented in square brackets, and the results are consistent with those of previous studies in showing that participants generally prefer the “risk averse” to the “risky” choices.

1. Positive monetary decision 1 (+Mon1), adapted from Tversky and Kahneman’s [2] Problem 3i.
  - Choose between:
  - A. A sure gain of HK$240 (66.04%)
  - B. 25% chance to gain HK$1,000, and
  - 75% chance to gain nothing (33.96%)
2. Positive monetary decision 2 (+Mon2), adapted from Tversky and Kahneman’s [2] Problem 5.
  - Choose between:
  - A. A sure win of HK$30 (55.66%)
  - B. 80% chance to win HK$45 (44.34%)
3. Positive medical decision (+Med 1), adapted from Tversky and Kahneman’s [2] Problem 1.
  - Choose between:
  - A. 200 people will be saved (60.38%)
  - B. 1/3 probability that 600 people will be saved, and 2/3 probability that no people will be saved (39.62%)

### Results and Discussion

We first analyzed the responses of the two monetary decisions (see Table 1). Among 66% (69 out of 105) of participants who chose the “risk averse” option in +Mon1, 57% (39 out of 69) chose the “risk averse” option again in +Mon2. Among 34% (36 out of 105) who chose the “risky” option in +Mon1, 53% (19 out of 36) chose the “risk averse” option in +Mon2. The percentages of choosing the “risk averse” option from the two groups of participants (i.e., 57% and 53%) were very close to the 56% (58 out of 105) of the overall percentage of the sample. These results indicate that a person’s choice of risk in one decision may not always predict his or her risk preference in another decision. Importantly, logistic regression showed that responses in +Mon1 accounted for 0% variance of responses in +Mon2, *Nagelkerke R^2^* = .00, *χ^2^ = *.13, *p* = .71.

**Table 1:** Results from Positively Framed Vignettes (crossing +Mon1 and +Mon2) in Experiment 1.

	+Mon1 “Risk Averse” Choice		+Mon1 “Risky” Choice	
	+Mon2 “Risk Averse”Choice	+Mon2 “Risky” Choice	+Mon2 “Risk Averse”Choice	+Mon2 “Risky” Choice
Number of participants	39	30	19	17

The above analysis tests the prediction from free will by testing a null hypothesis, which is logically not able to be confirmed. Alternatively, the findings could be analyzed from a perspective in which the prediction of complete determinism to be the null hypothesis and the free will prediction to be the alternative hypothesis. That is, a complete determinism suggests that people should exhibit 100% consistency between their responses in +Mon1 and +Mon2. Our results showed that only 55.24% (58 out of 105) showed this consistency, which is significantly smaller than 100%. A Chi square test that corrected the expected frequency of 100% to be including at least a cell with expected frequency of 5 (i.e., 100 vs. 5) was significant, χ^2^(1) = 370.44, *p*<.0001.

There was an issue of individual difference in terms of risk attitude that might complicate the interpretations of our findings. It is noteworthy that because the +Mon1 and +Mon2 were not identical, one problem might promote risk taking more than might the other problem. Therefore, it was possible that individuals with a different risk attitude (e.g., with some specific values on parameters of risk taking or risk aversion coefficients) would exhibit different preferences for the two problems, resulting in the unpredictability between +Mon1 and +Mon2. If the unpredictability was primarily due to this individual difference, then it means that the participants’ choice behaviors could still be predictable.

We checked this possibility with the following logic. Assume that one problem is more risk promoting than the other one, if participants’ risk preference is determined and hence showing consistency in their risk preference, then a couple of observations are expected. First, those who choose the risky option in the less risk promoting problem should be determined to choose the risky option in the more risk promoting problem, regardless of their risk attitude. Second, those who choose the risk averse option in the more risk promoting problem should be determined to choose the risk aversion option in the less risk promoting problem, again regardless of their risk attitude. We conducted the following analyses to examine these possibilities.

Assume that, due to whatever reasons, +Mon1 was more risk promoting than +Mon2. Those who were risk averse in +Mon1 were those who were very conservative. These participants should also be risk averse in +Mon2. There were 65.71% of participants (69 out of 105) who took the risk averse option in +Mon1. Among these 69 participants, 56.52% of participants (39 out of 69) took the risk averse option for +Mon2. The 56.52% of consistency was not significantly different from the 65.71% of the response distribution in +Mon1, χ^2^(1) = 2.59, *p*>.05, but was significantly different from 100%, corrected χ^2^(1) = 134.77, p<.0001. In addition, those who were risk taking in +Mon2 should also be risk taking in +Mon1. There were 44.76% (47 out of 105) who took the risk option in +Mon2. Among them 36.17% (17 out of 47) took the risk option in +Mon1, which was not different from 44.76%, χ^2^(1) = 1.40, *p*>.05, but was significantly different from 100%, corrected χ^2^(1) = 32.23, *p*<.0001.

Conversely, assume that, due to whatever reasons, +Mon2 was more risk promoting than +Mon1. Those who were risk averse in +Mon2 were those who were very conservative. These participants should also be risk averse in +Mon1. There were 55.24% of participants (58 out of 105) who took the risk averse option for +Mon2. Among these 58 participants, 67% (39 out of 58) took the risk averse option for +Mon1, which was not significantly different from 55.24%, χ^2^(1) = 2.97, *p*>.05, but was significantly different from 100%, corrected χ^2^(1) = 42.90, *p*<.0001. In addition, those who were risk taking in +Mon1 should also be risk taking in +Mon2. There were 34.29% (36 out of 105) took the risk option in +Mon1. Among them 47% (17 out of 36) took the risk option in +Mon2, which was not significantly different from 34.29%, χ^2^(1) = 2.67, *p*>.05, but was significantly different from 100%, corrected Chi = 33.45, *p*<.0001.

Finally, we also analyzed the predictability between +Mon1 and +Med1 (see Table 2) and between +Mon2 and +Med1 (Table 3). For +Mon1 predicting +Med1, logistic regression showed a non-significant *Nagelkerke R^2^* = .02, *χ^2^ = *1.18, *p* = .28. For +Mon2 predicting +Med1, logistic regression also showed a non-significant *Nagelkerke R^2^* = .01, *χ^2^ = *.78, *p* = .38.

**Table 2:** Results from Positively Framed Vignettes (crossing +Mon1 and +Med1) in Experiment 1.

	+Mon1 “Risk Averse” Choice		+Mon1 “Risky” Choice	
	+Med1 “Risk Averse”Choice	+Med1 “Risky” Choice	+Med1 “Risk Averse”Choice	+Med1 “Risky” Choice
Number of participants	44	25	19	17

**Table 3:** Results from Positively Framed Vignettes (crossing +Mon2 and +Med1) in Experiment 1.

	+Mon2 “Risk Averse” Choice		+Mon2 “Risky” Choice	
	+Med 1 “Risk Averse”Choice	+Med 1 “Risky” Choice	+Med 1 “Risk Averse”Choice	+Med 1 “Risky” Choice
Number of participants	37	21	26	21

In summary, results from Experiment 1 indicate that when information is positively framed, people’s risk preference in a decision does not predict their preference in another highly similar decision. These findings resemble the *Drosophila’s* behaviors demonstrated by previous studies [16], [17], suggesting that although the positively framed information and people’s genetic component jointly determine the probability of their choice behavior, people seem to *freely* determine their choices.

## Experiment 2

### Methods and Materials

The design of Experiment 2 (*N* = 96) was identical to that of Experiment 1, except that all the vignettes were negatively framed (i.e., described in terms of loss). The percentage of participants who chose each option is presented in square brackets; the results indicate that people generally prefer “risk” to “risk averse” options.

1. Negative monetary decision 1 (-Mon1), adapted from Tversky and Kahneman’s [2] Problem 3ii.
  - Choose between:
  - A. A sure loss of HK$750 (17.71%)
  - B. 25% chance to lose HK$1,000, and 75% chance to lose nothing (82.29%)
2. Negative monetary decision 2 (-Mon2), modified from Tversky and Kahneman’s [2] Problem 5.
  - Choose between:
  - A. A sure loss of HK$30 (40.63%)
  - B. 80% chance to lose HK$45 (59.38%)
3. Negative medical decision (-Med 1), adapted from Tversky and Kahneman’s [2] Problem 2.
  - Choose between:
  - A. 400 people will die (21.88%)
  - B. 1/3 probability that nobody will die, and 2/3 probability that 600 people will die. (78.13%)

### Results and Discussion

As done previously, we first analyzed the responses of the two monetary decisions (see Table 4). Among 18% (17 out of 96) of participants who chose the “risk averse” option in -Mon1, 65% (11 out of 17) chose the “risk averse” option again in -Mon2. Among 82% (79 out of 96) who chose the “risky” option in -Mon1, 35% (28 out of 79) chose the “risk averse” option in -Mon2. The percentages of choosing the “risk averse” option from the two groups of participants (i.e., 35% and 65%) were not that close to the 59% (57 out of 96) of the overall percentage of the entire sample. Such results indicate that a person’s choice of risk cannot predict his or her risk preference in another decision. Logistic regression showed that responses in -Mon1 significantly accounted for 7% variance of responses in -Mon2, *Nagelkerke R^2^* = .07, *χ^2^ = *4.89, *p* = .03. Furthermore, there were 60.42% of participants (58 out of 96) showed consistent preference between –Mon1 and –Mon2, which is significantly fewer than 100%, corrected *χ* ^2^(1) = 381.77, p<.0001.

**Table 4:** Results from Positively Framed Vignettes (crossing -Mon1 and -Mon2) in Experiment 2.

	−Mon1 “Risk Averse” Choice		−Mon1 “Risky” Choice	
	−Mon2 “Risk Averse”Choice	−Mon2 “Risky” Choice	−Mon2 “Risk Averse”Choice	−Mon2 “Risky” Choice
Number of participants	11	6	28	51

Next, we checked the issue of individual differences. Assume that, due to whatever reasons, -Mon1 was more risk promoting than -Mon2. Those who are risk averse in -Mon1 were those who were very conservative. That means, these participants should also be risk averse in -Mon2. There were 17.71% of participants (17 out of 96) took the risk averse option for -Mon1. Among these 17 participants 35.29% (6 out of 17) took the risk averse option for -Mon2., which was not significantly different from 17.71%, *χ* ^2^(1) = 3.61, *p*>.05. We could not examine whether it was significantly different from 100% because the number of cases was too few. In addition, those who were risk taking in -Mon1 should also be risk taking in -Mon2. There were 82.29% (79 out of 96) took the risk option in -Mon1. Among them 64.56% (51 out of 79) took the risk option in –Mon2, which was significantly fewer than 82.29%, *χ* ^2^(1) = 17.04, *p*<.01, and was also significantly fewer than 100%, corrected *χ* ^2^(1) = 112.95, p<.0001.

Conversely, assume that, due to whatever reasons, -Mon2 was more risk promoting than –Mon1. Those who were risk averse in -Mon2 were those who very conservative. That means, these participants should also be risk averse in -Mon1. There were 40.63% of participants (39 out of 96) took the risk averse option for -Mon2. Among these 39 participants 28.21% (11 out of 39) took the risk averse option for -Mon1, which was not significantly different from 40.63%, *χ* ^2^(1) = 2.39, *p*>.05, but was significantly fewer than 100%, corrected *χ* ^2^(1) = 8.26, p<.0001. In addition, those who were risk taking in -Mon2 should also be risk taking in -Mon1. There were 59.38% (57 out of 96) took the risky option in -Mon2. Among them 89.47% (51 out of 57) took the risk option for -Mon1, which was significant more than 59.38%, *χ* ^2^(1) = 21.40, *p*<.0001, but was not significantly fewer than 100%, corrected *χ* ^2^(1) = .22, *p*>.05.

Finally, we also analyzed the predictability between -Mon1 and -Med1 (see Table 5) as well as between -Mon2 and -Med1 (Table 6). Logistic regression for -Mon1 predicting -Med1 showed a non-significant *Nagelkerke R^2^* = .03, *χ^2^ = *1.99, *p* = .16. Logistic regression for +Mon2 predicting +Med1 also showed a non-significant *Nagelkerke R^2^* = .02, *χ^2^ = *1.52, *p* = .22.

**Table 5:** Results from Positively Framed Vignettes (crossing -Mon1 and –Med1) in Experiment 2.

	−Mon1 “Risk Averse” Choice		−Mon1 “Risky” Choice	
	−Med1 “Risk Averse”Choice	−Med1 “Risky” Choice	−Med1 “Risk Averse”Choice	−Med1 “Risky” Choice
Number of participants	6	11	15	64

**Table 6:** Results from Positively Framed Vignettes (crossing –Mon2 and –Med1) in Experiment 2.

	−Mon2 “Risk Averse” Choice		−Mon2 “Risky” Choice	
	−Med 1 “Risk Averse”Choice	−Med 1 “Risky” Choice	−Med 1 “Risk Averse”Choice	−Med 1 “Risky” Choice
Number of participants	11	28	10	47

In summary, results from Experiment 2 indicate that when information is negatively (vs. positively) framed, people exhibit a more consistent risk preference across different risk decisions. However, the predictability from a person’s response in one decision to another decision is very low. The greatest predictability has been observed between -Mon1 and -Mon2, in which only 7% of variances are shared. Also, the analyses of the issue of individual difference showed no evidence of determinism, except that if –Mon2 was more risk promoting than –Mon1, those who took the risky option in –Mon2 were consistently took the risky option in –Mon1. Thus, although the result is not as extreme as that of Experiment 1, a person’s risk preference in negatively framed conditions is still characterized by substantial variations.

### Conclusions

In this research, we pose a question that explores the extent to which a person’s choice could be a (free) choice. Replicating the findings of Tversky and Kahneman, people’s choice behavior is largely determined by how information is framed: the tendency to be risk averse in positively framed situations, and the tendency to be risk seeking in negatively framed situations. This research extended the body of classic studies by testing how likely people exhibit the same risk preference across different situations with similar risk implications. Consistent with previous research on *Drosophila*, the present study reveals that all personal and situational factors determine only the *probability* of choice behaviors, and a large room of variations exists in which a person may freely choose.

As stated in the Introduction, demonstrating the presence of variations does not indicate the existence of free will because randomness can also produce the same variations [8]. Some scholars doubt that separating randomness effects from free will effects is a scientific research topic [4]. Nonetheless, this variation indicates the existence of a considerable room of variations that may allow a person to have free will in their choice behaviors.
